# Relapse Prevention in Acute Myeloid Leukemia: The Role of Immunotherapy with Histamine Dihydrochloride and Low-Dose Interleukin-2

**DOI:** 10.3390/cancers16101824

**Published:** 2024-05-10

**Authors:** Pau Montesinos, Francesco Buccisano, Thomas Cluzeau, Lovisa Vennström, Michael Heuser

**Affiliations:** 1 Hematology Department, La Fe University and Polytechnic Hospital, 46026 Valencia, Spain; montesinos_pau@gva.es; 2 Department of Biomedicine and Prevention, University of Rome Tor Vergata, 00133 Rome, Italy; francesco.buccisano@uniroma2.it; 3 Department of Hematology, University Hospital Centre of Nice, 06200 Nice, France; cluzeau.t@chu-nice.fr; 4 Department of Hematology and Coagulation, Sahlgrenska University Hospital, 41345 Goteborg, Sweden; lovisa.vennstrom@vgregion.se; 5 Department of Hematology, Hemostasis, Oncology and Stem Cell Transplantation, Hannover Medical School, 30625 Hannover, Germany

**Keywords:** acute myeloid leukemia, maintenance, immunotherapy, histamine, interleukin-2

## Abstract

**Simple Summary:**

The treatment of acute myeloid leukemia (AML) has improved in recent decennia by targeted therapy and allogeneic stem cell transplantation (allo-SCT). Hematological relapse in patients who have attained complete remission (CR) after chemotherapy remains a significant cause of mortality, so remission maintenance in AML is a major challenge. Maintenance with histamine dihydrochloride associated with low-dose interleukin-2 (HDC/LD-IL-2), both administered by subcutaneous injections, has been approved for patients in CR who are not candidates for upfront allo-SCT. Clinical studies showed that HDC/LD-IL-2 significantly and sustainably improved leukemia-free survival with a good safety profile, and that patients less than 60 years old in CR after one induction and/or with a normal karyotype would particularly benefit from the association. The immunomodulatory action of HDC/LD-IL-2 is mediated by the expansion of the NK cell population and a concomitant improvement in NK cell functionality. Immunotherapy with HDC/LD-IL-2 may represent an emerging treatment option for remission maintenance in AML.

**Abstract:**

The treatment and management of acute myeloid leukemia (AML) has improved in recent decennia by targeted therapy for subgroups of patients, expanded indications for allogeneic stem cell transplantation (allo-SCT) and surveillance of residual or arising leukemia. However, hematological relapse among patients who have attained complete remission (CR) after the initial courses of chemotherapy remains a significant cause of morbidity and mortality. Here, we review an immunotherapeutic option using histamine dihydrochloride and low-dose interleukin-2 (HDC/LD-IL-2) for remission maintenance in AML. The treatment is approved in Europe in the post-consolidation phase to avoid relapse among patients in CR who are not candidates for upfront allo-SCT. We present aspects of the purported anti-leukemic mechanism of this regimen, including translation of preclinical results into the clinical setting, along with relapse prevention in subgroups of patients. We consider that HDC/LD-IL-2 is a conceivable option for younger adults, in particular patients with AML of normal karyotype and those with favorable responses to the initial chemotherapy. HDC/LD-IL-2 may form an emerging landscape of remission maintenance in AML.

## 1. Introduction

Although several initial options are available, the standard initial treatment for adult patients with acute myeloid leukemia (AML) is the combination of cytarabine and anthracyclines followed by repeated courses of consolidation [1,2,3,4]. This scheme of treatment has been used for several decades and was recently supplemented with FTL3-inhibitors during induction and consolidation courses for patients with FLT3-mutant AML [2,3,5]. However, relapses in CR are common and represent a leading cause of morbidity and death [6,7]. Allogeneic stem cell transplantation (allo-SCT) may be included in the early phase of therapy for patients with intermediate- or high-risk cytogenetics or in patients who do not achieve CR with the initial induction therapy but is not recommended in patients with favorable risk, such as nucleophosmin-1 (NPM1)-mutated or core-binding factor AML [2,3,4,8]. While allo-SCT reduces relapse risk, transplant-related morbidity and mortality outweigh the benefit of transplantation in patients at lower risk of relapse.

The current guidelines of the European LeukemiaNet (ELN) recommend two drugs in the post-complete-remission (CR) phase of AML: the FLT3 inhibitor midostaurin (for patients with FLT3-mutated AML) and oral azacitidine (with documented efficacy in older patients) [3]. For younger adults who are not candidates for allo-SCT, there is no consensually efficacious relapse-preventive strategy available. Improved maintenance therapy, defined as extended treatment beyond the initial chemotherapy to avoid relapse in CR or prolong the duration of remission, is thus a partly unmet need in AML [2,3,4].

Immunotherapy with histamine dihydrochloride (HDC) and low-dose interleukin-2 (LD-IL-2) was developed in the 1980s to improve anti-leukemic functions of natural killer (NK) cells [9,10]. HDC/LD-IL-2 was evaluated as maintenance immunotherapy in a phase 3 study in 320 AML patients in the post-consolidation phase [11]. The trial met the primary endpoint of improved leukemia-free survival (LFS) and HDC was approved by the European Medicines Agency as maintenance therapy in AML in conjunction with LD-IL-2 for adult patients in first CR (CR1). More recently, the results of phase 3 were further analyzed to identify biomarkers of improved outcome [12,13,14]. Here, we review the proposed mechanism of action along with the potential value of HDC/LD-IL-2 for relapse prevention with emphasis on responsive patient profiles, including the age, karyotype, and chemosensitivity of leukemic cells.

## 2. HDC/LD-IL-2 for Remission Maintenance: Rationale and Results

### 2.1. Immune Activation by HDC/LD-IL-2

Results in preclinical models imply that a primary mode of action of HDC/LD-IL-2 is the activation of NK cell function, including induction of the expression of natural cytotoxicity receptors (NCRs) such as NKp30 and NKp46 [12,15]. A principal aspect of HDC/LD-IL-2 therapy is the targeting of immunosuppressive myeloid cells. The NADPH oxidase 2 (NOX2) expressed by myeloid cells (including mature monocytic AML cells) generates highly immunosuppressive reactive oxygen species (ROS) that inhibit the expression of NCRs, including NKp46 and NKp30, and also trigger the apoptosis of NK cells to impede their cytolytic activity against leukemic cells [16,17,18,19,20]. The histamine H2 receptor (H2R) is co-expressed with NOX2 on normal and leukemic monocytic cells. Agonist activity at H2R thus inhibits ROS formation from NOX2 and restores antileukemic functions of NK cells as well as NKp46 and NKp30 receptor expression [10,12]. By this mechanism, HDC or similar compounds with agonist activity at H2 receptors reduce the expansion of myeloid leukemia cells in murine models in vivo [21] and synergize with IL-2 to trigger the NK-cell-dependent lysis of primary AML cells [10,22].

In attempts to translate these findings into the clinical setting, NK cell activation by HDC/LD-IL-2 was assessed in serial blood samples of AML patients in CR who received HDC/LD-IL-2 in a phase 4 trial. It was shown that the 3-week cycles of therapy entailed expansion of NK cells along with upregulation of NKp30 and NKp46 receptor expression on individual NK cells [12]. In the phase 4 trial, upregulation of NK cell receptors during the initial cycles of HDC/LD-IL-2 was significantly correlated with reduced relapse risk, thus suggesting that NK cell activation contributes to the anti-leukemic efficacy of this regimen, as further discussed below.

A potential direct role of HDC-LD-IL-2 on T cells may also be considered. Studies in murine cancer models known to entail accumulation of myeloid-derived suppressor cells (MDSC) support the capacity of HDC-LD-IL-2 to promote effector functions of tumor-infiltrating CD8+ T cells [23]. In these experiments, MDSCs isolated from HDC-treated mice had a reduced capacity to suppress CD8+ T cell proliferation, which supports the capacity of HDC to target a significant effector function in MDSC-mediated immunosuppression. On the other hand, IL-2 is known as a central regulator of immune responses by balancing the suppression of immune responses through the maintenance of regulatory T cells (Tregs) and the induction of immune responses through the proliferation and differentiation of effector T cells [24]. The highest constitutive expression of the IL-2Rα receptor (IL-2Rα/CD25) is found on Treg (CD4+/CD25+) cells. However, while the expression on effector T cells is low at a resting state, it is dramatically upregulated upon activation of the T-cell receptor, which may contribute to expanding tumor-specific CD8+/CD25+ T cells and to a better antitumor efficacy [25].

### 2.2. HDC/LD-IL-2: Results of the Pivotal Phase 3 Study

The clinical benefit of HDC/LD-IL-2 for maintenance in AML was evaluated in a phase 3 study including 320 adult patients from Australia, Canada, Europe, Israel, New Zealand, and the United States [11]. Patients in CR were randomly assigned after the completion of post-remission chemotherapy to receive HDC at 0.5 mg and IL-2 at 16,400 U/kg (one subcutaneous [s.c.] injection of each compound bid) or no treatment (standard of care).

The choice of dosing (cycles of 3-week HDC/LD-IL-2 s.c. with 3-week [cycles 1–3] or 6-week [cycles 4–10] treatment-free periods during 18 months) was a compromise between the immune activation and tolerability of IL-2, resulting in a dose of IL-2 that is 40-fold lower than that used in oncology to treat renal cell carcinoma or other solid cancers [26]. The lower dose of IL-2 also allowed for unsupervised home treatment by patients after brief instruction [22]. The dose of HDC was chosen to achieve >70% saturation of H2R for at least 4 h. Exclusion criteria were previous allo-SCT, unstable cardiac disease, active peptic ulcer and history of recent asthma. The median age of enrolled patients was 55 years (range 18–84). Sixty percent of the randomly assigned patients were <60 years old, and eighty percent were in CR1. All patients were followed for relapse and survival for >3 years (median: 48 months).

The phase 3 trial reached the primary endpoint of improved LFS in all randomly assigned patients (n = 320, hazard ratio [HR] 0.71, *p* = 0.008) and the secondary endpoint of LFS in patients in CR1 (n = 261, HR 0.69, *p* < 0.01) (Table 1) [11]. The primary endpoint remained significantly in favor of HDC/LD-IL-2 in multivariable analysis, taking potential confounders into account (adjusted *p*-value 0.006 in patients < 60 years with a normal karyotype). The trial did not reach the secondary endpoint of overall survival (OS), albeit with a trend toward improved survival in patients < 60 years in CR1 (HR 0.65, *p* = 0.07). Within the group of patients receiving HDC/LD-IL-2, the HR of LFS and OS were strikingly correlated (R^2^ = 0.88–0.93) within participating countries, thus supporting that LFS was a valid surrogate marker for OS [27].

The frequency of grade 3 and 4 events did not differ between study arms with no treatment-related mortality. The most prevalent side-effects in the treatment arm were transient flush, mild hypotension, headache (all likely attributed to the HDC component), transient and mild thrombocytopenia, transient eosinophilia, low-grade fever, injection site reactions to IL-2, nausea and dyspepsia. While 92% of patients who remained in CR took the treatment as prescribed, transient dose reductions were required in 26% of patients, mostly during the initial cycles of therapy. Serial assessment of quality-of-life (QoL) using the EORTC-QL40 instrument showed that patients in both study arms increased or maintained their QoL status from baseline to last evaluation with respect to global health status as well as fatigue, nausea/vomiting, pain, diarrhea, and dyspnea. None of these aspects of health were negatively impacted by treatment [28]. In 2008, HDC/LD-IL-2 became the first immunotherapy approved as maintenance therapy in AML in the European Union, with confirmed approval in 2018.

### 2.3. Immunomodulatory Action of HDC/LD-IL-2: Results of the Re:Mission Phase 4 Study

The objective of the subsequent Re:Mission one-armed phase 4 study was to illuminate immunomodulation during treatment with HDC/LD-IL-2 and to define immune cell subsets that may predict clinical outcome [12]. Eligible patients (n = 84, age 18–79, all in CR1) received up to ten 3-week cycles of HDC/LD-IL-2, thus duplicating the regimen of the phase 3 trial. Peripheral blood mononuclear cells were collected before and after cycles 1 and 3. The treatment induced increments in NK cell counts in blood during cycles (3-fold increase during cycle 1) along with the upregulation of natural cytotoxicity receptors (NKp30 and NKp46) expressed by NK cells, suggesting that HDC/LD-IL-2 expanded the NK cell population and concomitantly improved NK cell functionality.

In patients included in the phase 4 study, the impact of the dynamics of cytotoxic T-cell phenotypes on LFS or OS was also assessed. It was shown that transition from memory to effector T cells during cycle 1 as well as the activation of NK cells (assessed by NKp46 receptor expression) were both associated with improved outcome. Patients achieving the induction of NCR along with the transition of T memory to effector cells were strikingly protected from relapse (85% LFS at >2 years) and death (100% OS at >2 years) [12]. Regarding the expected role of IL-2 on Tregs, further analyses of serial blood samples from patients participating in the phase IV Re:Mission trial showed that Tregs were induced during initial cycles of HDC/IL-2 and that the induction diminished in subsequent treatment cycles, with no impact on relapse risk [29]. These data do not support Treg targeting for additionally efficacious immunotherapy with HDC/IL-2.

The Re:Mission trial results thus suggest that activation of anti-leukemic lymphocytes contributed to the clinical benefit of HDC/LD-IL-2. Additionally, patients receiving HDC/LD-IL-2 showed a 60–70% reduction in blood counts of NOX2-expressing myeloid-derived suppressor cells (MDSCs) during the first cycle of therapy. The magnitude of reduction in MDSCs significantly correlated with reduced relapse risk, thus supporting that HDC/LD-IL-2 targets aspects of immunosuppression during therapy [23].

### 2.4. Post Hoc Analyses According to Chemosensitivity and Karyotype

**Figure 1:**
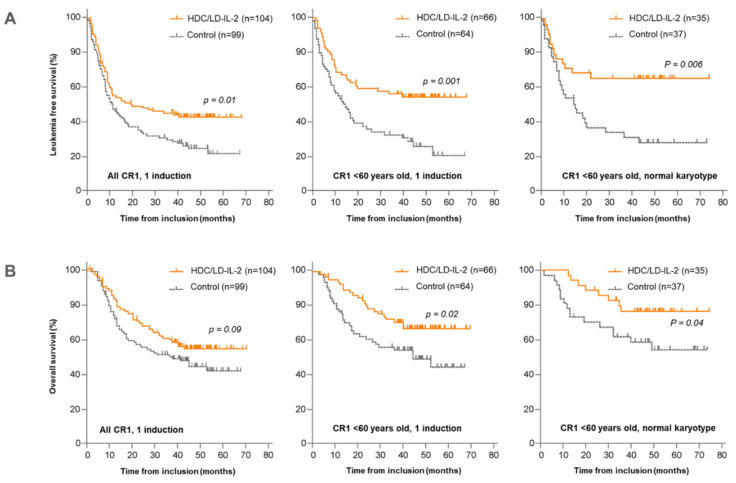
Effect of HDC/LD-IL-2 as AML maintenance therapy on leukemia-free and overall survival according to CR status, age and karyotype in patients not eligible for allo-SCT. The effects of HDC/LD-IL-2 have been assessed in post hoc analyses on (**A**) leukemia free survival and (**B**) overall survival. The results are presented for all CR1 (left panel) or less-than-60-years-old (middle panel) patients achieving CR1 after only one induction. The results according to normal karyotype are presented for CR1 patients less than 60 years old (right panels). Adapted from phase 3 post hoc analyses [13,14].

The requirement of more than one cycle of induction chemotherapy to achieve CR is a risk factor for relapse and death in younger adult patients [30]. A post hoc analysis evaluated the benefit of HDC/LD-IL-2 according to the number of inductions (one or more) and age (< or ≥60 yrs). A preferential clinical benefit of HDC/LD-IL-2 was observed in patients < 60 years old having achieved CR1 after one course of induction (“chemosensitive AML”, HR 0.46, *p* < 0.001) (Figure 1 and Table 1). In this subgroup, 3-year OS on HDC/LD-IL-2 was improved in the treatment arm (HR 0.53, *p* = 0.02). Patients > 60 years old with chemosensitive AML did not significantly benefit from HDC/LD-IL-2 in terms of LFS or OS (*p* > 0.4). Failure to achieve CR1 after one induction course was observed in 22% of patients and largely abated the benefit of HDC/LD-IL-2 [13].

The phase 3 study was initiated when AML was categorized mainly by cytogenetics and FAB classification. Normal-karyotype AML comprises mutations in *NPM1*, *FLT3-ITD*, *CEBPA*, *DNMT3A*, *IDH1* and *IDH2*. Mutations of *NPM1* or *FLT3-ITD*, alone or in combination, are the most prevalent mutations and account for approximately 40–50% and 30–40% of cases, respectively. Post hoc analysis based on data available from 225 patients enrolled in phase 3 showed a benefit of HDC/LD-IL-2 on LFS and OS in patients harboring leukemic cells of normal karyotype [14]. The benefit of HDC/LD-IL-2 on LFS was pronounced in patients < 60 years old with normal-karyotype AML (HR 0.40, LFS, *p* = 0.006). Results in this subgroup suggest durable prevention against relapse (Figure 1 and Table 1). HDC/LD-IL-2 did not benefit patients with aberrant karyotypes or older patients with a normal karyotype [14].

## 3. Additional Approved Options in AML Maintenance Therapy

HDC/LD-IL-2 was not available, while being approved, at the time when the ELN guidelines were published [3]. Those guidelines considered two options in maintenance therapy: midostaurin and oral azacitidine.

The FLT3 inhibitor midostaurin is approved for maintenance therapy in patients with FLT3-mutated AML who remain in CR after induction and consolidation chemotherapy. The approval and recommendation of midostaurin in AML maintenance therapy were based on the RATIFY phase 3 trial that evaluated midostaurin added during induction and consolidation chemotherapy [31]. Patients in CR after consolidation entered a maintenance phase to receive midostaurin or placebo [32]. The results showed significantly prolonged OS and disease-free survival in the treatment arm. The relative benefit of midostaurin in the maintenance phase could not be distinguished from effects during induction and consolidation [1,2,33,34]. In the RATIFY trial, a long-term benefit in terms of OS was observed for patients in the treatment arm, supporting the curative impact of FLT3 inhibition. More recently, quizartinib, a selective FLT3-ITD type 2 inhibitor, showed a benefit in terms of OS in a phase 3 trial for patients with FLT3-ITD^+^ AML (QUANTUM-First trial) [35]. Like midostaurin, quizartinib was included during induction and consolidation and patients remaining in CR continued quizartinib or placebo. Quizartinib is approved by EMA and FDA for use during induction and consolidation and in the post-chemotherapy phase. Overall, these trials highlight the benefit of FLT3 inhibitors in FLT3^+^ AML, although the impact of FLT3 inhibition in the post-remission phase should be further elucidated. A recent placebo-controlled randomized study in patients receiving gilteritinib after allo-SCT suggests a possible benefit of FLT3 inhibitors in this setting for patients with detectable FLT3-ITD MRD pre- or post-HCT [36].

Oral azacitidine is approved for maintenance therapy in AML patients who have achieved CR, or CR with incomplete blood count recovery, following induction therapy with or without consolidation treatment and who are not candidates for allo-SCT. The approval is based on results of the QUAZAR phase 3 study showing significant improvement in OS and relapse-free survival in the treatment arm [37]. In primary analysis, median OS was 24.7 months in the oral azacitidine group vs. 14.8 months in controls (*p* < 0.001). Patients with favorable prognosis according to the 2011 classification of the National Comprehensive Cancer Network (NCCN) Clinical Practice Guidelines in AML [38] and patients < 55 years old were not included in this trial [37]. Post hoc analyses showed that the effect on survival was pronounced in NPM1-mutated AML [39]. Approximately two-thirds of patients included received one or two consolidation cycles, and extrapolation of efficacy into more intensive schemes of consolidation remains to be established. In the QUAZAR study, the percentages of patients who discontinued for adverse events were 13% in the oral azacitidine group and 4% in the placebo group [37]. Half of patients receiving oral azacitidine experienced drug-related diarrhea [37], mostly during early cycles of treatment. However, oral azacitidine did not negatively impact overall aspects of quality of life [40].

## 4. Conclusions

HDC/LD-IL-2 is an immunotherapeutic strategy to reduce the risk of relapse in the post-remission phase of AML. The clinical efficacy of this regimen likely derives from countering myeloid-cell-induced immunosuppression (HDC component) along with the activation of anti-leukemic lymphocytes (NK cells, cytotoxic T cells; IL-2 component), although additional or supplementary effects are conceivable. According to post hoc analyses of phase 3 trial results, HDC durably reduces relapse risk among patients with AML of normal karyotype and in patients with leukemic cells responsive to initial chemotherapy; in these subgroups, treatment with HDC/LD-IL-2 largely doubles the likelihood of long-term relapse-free survival with a similar magnitude of efficacy for overall survival. The reason for the preferential clinical efficacy in normal-karyotype AML remains unknown but may point toward specific mutational targets within this patient group and/or a variable impact of cell-mediated immunity between subtypes of AML. The clinical benefit of HDC/LD-IL-2 is age-dependent, with preferred efficacy in younger patients (<60 years old). In this regard, HDC/LD-IL-2 may provide an option for maintenance treatment alongside oral azacitidine, which hitherto has only been evaluated for relapse prevention in older patients.

The self-administration by subcutaneous injection may be perceived as a hurdle to the wide-spread use of HDC/LD-IL-2. While the future development of orally available derivatives of HDC and slow-release variants of IL-2 will likely improve the uptake of this regimen, it should be emphasized that >90% of patients remaining in CR in phase 3 took the study drugs as prescribed with no recorded impact on aspects of global health during therapy. Notably, the duration of therapy is limited to 18 months, during which patients take treatment for 30 out of 78 weeks. Additionally, phase 2 results report that >70% of patients on HDC/LD-IL-2 could return to gainful employment within the first year after initiating therapy, suggesting that treatment does not significantly hinder daily life activities.

## 5. Future Directions

HDC/LD-IL-2 reduces relapse risk in younger adult patients who are not candidates for upfront allo-SCT. For these patients, there is no consensually accepted alternative therapy apart from FLT3 inhibitors in FLT3^+^ AML. Future studies should address the detailed impact of HDC/LD-IL-2 in subgroups of AML, including efficacy according to MRD status after completed chemotherapy and effects of HDC/LD-IL-2 on the switch of MRD to positivity or negativity. Such studies would facilitate the alignment of HDC/LD-IL-2 with current AML classification and recommendations. Additionally, the optimal duration of therapy should be further clarified: according to phase 3 results achieved in, e.g., a normal karyotype, relapses were uncommon after 9–12 months of therapy, suggesting that a shorter treatment duration is conceivable.

The clinical benefit of HDC/LD-IL-2 in AML may inspire further development of immunotherapeutic strategies for remission maintenance in AML, including alternative strategies to target myeloid-cell-related immunosuppression and/or alternative means to achieve the optimal activation of anti-leukemic lymphocytes. The immunological action of HDC/LD-IL-2 in the post-transplant setting deserves to be assessed.

## Figures and Tables

**Table 1:** Main clinical results with HDC/LD-IL-2 in AML maintenance therapy after intensification and consolidation with intensive chemotherapy.

Type of Study	Objective	Population Assessed	Main Results
Phase 3 [11]	Effect on LFS stratified according to CR (CR1 or >1) and safety	320 AML adults (18–84 yrs-old) with CR	- Significantly improved LFS vs. controls (40% vs. 26%; HR * 0.74) - Higher improvement in CR1 patients - AEs mild to moderate
Phase 4 [12]	Effect on NK cell induction and activation, and correlation with LFS	62 patients treated by HDC/LD-IL-2 for 18 months after induction and consolidation	- 3-fold increase in the absolute number of blood NK cells during cycle 1 - Induction of NCR on NK cells - Significant relationship between NK cell activation and LFS
Phase 3 post hoc analysis [13]	Evaluation of LFS according to the number of inductions (1 or more) and age (< or ≥60 yrs)	320 patients included in the phase 3 study	- Effect on LFS increased in patients with CR1 after 1 induction (HR 0.64) - Effect further increased in patients < 60 yrs old (HR 0.46) - No effect in older patients or in patients with CR1 after >1 induction
Exploratory analysis [14]	LFS according to normal vs. aberrant karyotype	Analyses from 84 patients (44 with normal karyotype) included in phases 3 and 4 trials	- Significant benefit on LFS only in CR1 patients with normal karyotype (HR 0.40) - Benefit further increased in patients < 60 yrs old

* Hazard ratio treated vs. no treatment.

## References

[1] Sekeres M.A., Guyatt G., Abel G., Alibhai S., Altman J.K., Buckstein R., Choe H., Desai P., Erba H., Hourigan C.S. (2020). American Society of Hematology 2020 guidelines for treating newly diagnosed acute myeloid leukemia in older adults. Blood Adv..

[2] Heuser M., Ofran Y., Boissel N., Brunet Mauri S., Craddock C., Janssen J., Wierzbowska A., Buske C. (2020). ESMO Guidelines Committee. Acute myeloid leukaemia in adult patients: ESMO Clinical Practice Guidelines for diagnosis, treatment and follow-up. Ann. Oncol..

[3] Döhner H., Wei A.H., Appelbaum F.R., Craddock C., DiNardo C.D., Dombret H., Ebert B.L., Fenaux P., Godley L.A., Hasserjian R.P. (2022). Diagnosis and management of AML in adults: 2022 recommendations from an international expert panel on behalf of the ELN. Blood.

[4] Roloff G.W., Odenike O., Bajel A., Wei A.H., Foley N., Uy G.L. (2022). Contemporary Approach to Acute Myeloid Leukemia Therapy in 2022. Am. Soc. Clin. Oncol. Educ. Book.

[5] Kantarjian H., Kadia T., DiNardo C., Daver N., Borthakur G., Jabbour E., Garcia-Manero G., Konopleva M., Ravandi F., Ravandi F. (2021). Acute myeloid leukemia: Current progress and future directions. Blood Cancer J..

[6] National Cancer Institute Surveillance, Epidemiology, and End Results Program. Cancer Stat Facts: Leukemia—Acute Myeloid Leukemia (AML). https://seer.cancer.gov/statfacts/html/amyl.html.

[7] Forman S.J., Rowe J.M. (2013). The myth of the second remission of acute leukemia in the adult. Blood.

[8] Dholaria B., Savani B.N., Hamilton B.K., Oran B., Liu H.D., Tallman M.S., Ciurea S.O., Holtzman N.G., Ii G.L.P., Devine S.M. (2021). Hematopoietic Cell Transplantation in the Treatment of Newly Diagnosed Adult Acute Myeloid Leukemia: An Evidence-Based Review from the American Society of Transplantation and Cellular Therapy. Transplant. Cell. Ther..

[9] Hellstrand K., Hermodsson S. (1986). Histamine H2-receptor-mediated regulation of human natural killer cell activity. J. Immunol..

[10] Martner A., Thorén F.B., Aurelius J., Hellstrand K. (2013). Immunotherapeutic strategies for relapse control in acute myeloid leukemia. Blood Rev..

[11] Brune M., Castaigne S., Catalano J., Gehlsen K., Ho A.D., Hofmann W.K., Hogge D.E., Nilsson B., Or R., Romero A.I. (2006). Improved leukemia-free survival after postconsolidation immunotherapy with histamine dihydrochloride and interleukin-2 in acute myeloid leukemia: Results of a randomized phase 3 trial. Blood.

[12] Martner A., Rydström A., Riise R.E., Aurelius J., Anderson H., Brune M., Foà R., Hellstrand K., Thorén F.B. (2015). Role of natural killer cell subsets and natural cytotoxicity receptors for the outcome of immunotherapy in acute myeloid leukemia. Oncoimmunology.

[13] Nilsson M.S., Hallner A., Brune M., Nilsson S., Thorén F.B., Martner A., Hellstrand K. (2020). Complete remission after the first cycle of induction chemotherapy determines the clinical efficacy of relapse-preventive immunotherapy in acute myeloid leukaemia. Br. J. Haematol..

[14] Nilsson M.S., Hallner A., Brune M., Nilsson S., Thorén F.B., Martner A., Hellstrand K. (2020). Immunotherapy with HDC/LD-IL-2 may be clinically efficacious in acute myeloid leukemia of normal karyotype. Hum. Vaccin. Immunother..

[15] Fauriat C., Just-Landi S., Mallet F., Arnoulet C., Sainty D., Olive D., Costello R.T. (2007). Deficient expression of NCR in NK cells from acute myeloid leukemia: Evolution during leukemia treatment and impact of leukemia cells in NCRdull phenotype induction. Blood.

[16] Hellstrand K., Asea A., Dahlgren C., Hermodsson S. (1994). Histaminergic regulation of NK cells. Role of monocyte-derived reactive oxygen metabolites. J. Immunol..

[17] Hansson M., Asea A., Ersson U., Hermodsson S., Hellstrand K. (1996). Induction of apoptosis in NK cells by monocyte-derived reactive oxygen metabolites. J. Immunol..

[18] Hansson M., Asea A., Hermodsson S., Hellstrand K. (1996). Histaminergic regulation of NK-cells: Protection against monocyte-induced apoptosis. Scand. J. Immunol..

[19] Gabrilovich D.I., Nagaraj S. (2009). Myeloid-derived suppressor cells as regulators of the immune system. Nat. Rev. Immunol..

[20] Grauers Wiktorin H., Aydin E., Hellstrand K., Martner A. (2020). NOX2-Derived Reactive Oxygen Species in Cancer. Oxid. Med. Cell. Longev..

[21] Aydin E., Hallner A., Grauers Wiktorin H., Staffas A., Hellstrand K., Martner A. (2019). NOX2 inhibition reduces oxidative stress and prolongs survival in murine KRAS-induced myeloproliferative disease. Oncogene.

[22] Brune M., Hellstrand K. (1996). Remission maintenance therapy with histamine and interleukin-2 in acute myelogenous leukaemia. Br. J. Haematol..

[23] Grauers Wiktorin H., Nilsson M.S., Kiffin R., Sander F.E., Lenox B., Rydström A., Hellstrand K., Martner A. (2019). Histamine targets myeloid-derived suppressor cells and improves the anti-tumor efficacy of PD-1/PD-L1 checkpoint blockade. Cancer Immunol. Immunother..

[24] Lokau J., Petasch L.M., Garbers C. (2024). The soluble IL-2 receptor α/CD25 as a modulator of IL-2 function. Immunology.

[25] Wu W., Chia T., Lu J., Li X., Guan J., Li Y., Fu F., Zhou S., Feng Y., Deng J. (2023). IL-2Rα-biased agonist enhances antitumor immunity by invigorating tumor-infiltrating CD25 + CD8+ T cells. Nat. Cancer.

[26] Dutcher J.P., Schwartzentruber D.J., Kaufman H.L., Agarwala S.S., Tarhini A.A., Lowder J.N., Atkins M.B. (2014). High dose interleukin-2 (Aldesleukin)—Expert consensus on best management practices-2014. J. Immunother. Cancer.

[27] Buyse M., Michiels S., Squifflet P., Lucchesi K.J., Hellstrand K., Brune M.L., Castaigne S., Rowe J.M. (2011). Leukemia-free survival as a surrogate end point for overall survival in the evaluation of maintenance therapy for patients with acute myeloid leukemia in complete remission. Haematologica.

[28] Wallhult E., Whisnant J., Rowe J.M., Szer J., Bhagwat D., Hellstrand K., Nilsson B.I., Brune M.L. (2007). Impact on Quality of Life of Postconsolidation Immunotherapy with Histamine Dihydrochloride and Interleukin-2 in Acute Myelogenous Leukemia. Blood.

[29] Sander F.E., Nilsson M., Rydström A., Aurelius J., Riise R.E., Movitz C., Bernson E., Kiffin R., Ståhlberg A., Brune M. (2017). Role of regulatory T cells in acute myeloid leukemia patients undergoing relapse-preventive immunotherapy. Cancer Immunol. Immunother..

[30] Othus M., Estey E.H., Garcia-Manero G., Wood B.L., Stirewalt D.L., Godwin J.E., Weick J.K., Anderson J.E., Appelbaum F.R., Erba H.P. (2019). Second cycle remission achievement with 7 + 3 and survival in adults with newly diagnosed acute myeloid leukemia: Analysis of recent SWOG trials. Leukemia.

[31] Larson R.A., Mandrekar S.J., Huebner L.J., Sanford B.L., Laumann K., Geyer S., Bloomfield C.D., Thiede C., Prior T.W., Döhner K. (2021). Midostaurin reduces relapse in FLT3-mutant acute myeloid leukemia: The Alliance CALGB 10603/RATIFY trial. Leukemia.

[32] Stone R.M., Mandrekar S.J., Sanford B.L., Laumann K., Geyer S., Bloomfield C.D., Thiede C., Prior T.W., Döhner K., Marcucci G. (2017). Midostaurin plus Chemotherapy for Acute Myeloid Leukemia with a FLT3 Mutation. N. Engl. J. Med..

[33] Reville P.K., Kadia T.M. (2021). Maintenance Therapy in AML. Front. Oncol..

[34] Schmalbrock L.K., Dolnik A., Cocciardi S., Sträng E., Theis F., Jahn N., Panina E., Blätte T.J., Herzig J., Skambraks S. (2021). Clonal evolution of acute myeloid leukemia with FLT3-ITD mutation under treatment with midostaurin. Blood.

[35] Erba H.P., Montesinos P., Kim H.J., Patkowska E., Vrhovac R., Žák P., Wang P.N., Mitov T., Hanyok J., Kamel Y.M. (2023). QuANTUM-First Study Group. Quizartinib plus chemotherapy in newly diagnosed patients with FLT3-internal-tandem-duplication-positive acute myeloid leukaemia (QuANTUM-First): A randomised, double-blind, placebo-controlled, phase 3 trial. Lancet.

[36] Levis M.J., Hamadani M., Logan B., Jones R.J., Singh A.K., Litzow M., Wingard J.R., Papadopoulos E.B., Perl A.E., Soiffer R.J. (2024). BMT-CTN 1506/MORPHO Study Investigators. Gilteritinib as Post-Transplant Maintenance for Acute Myeloid Leukemia With Internal Tandem Duplication Mutation of FLT3. J. Clin. Oncol..

[37] Wei A.H., Döhner H., Pocock C., Montesinos P., Afanasyev B., Dombret H., Ravandi F., Sayar H., Jang J.H., Porkka K. (2020). QUAZAR AML-001 Trial Investigators. Oral Azacitidine Maintenance Therapy for Acute Myeloid Leukemia in First Remission. N. Engl. J. Med..

[38] O’Donnell M.R., Abboud C.N., Altman J., Appelbaum F.R., Arber D.A., Attar E., Borate U., Coutre S.E., Damon L.E., Goorha S. (2012). NCCN Clinical Practice Guidelines Acute myeloid leukemia. J. Natl. Compr. Canc. Netw..

[39] Döhner H., Wei A.H., Roboz G.J., Montesinos P., Thol F.R., Ravandi F., Dombret H., Porkka K., Sandhu I., Skikne B. (2022). Prognostic impact of NPM1 and FLT3 mutations in patients with AML in first remission treated with oral azacitidine. Blood.

[40] Roboz G.J., Döhner H., Pocock C., Dombret H., Ravandi F., Jang J.H., Selleslag D., Mayer J., Martens U.M., Liesveld J. (2021). Oral azacitidine preserves favorable level of fatigue and health-related quality of life for patients with acute myeloid leukemia in remission: Results from the phase 3, placebo-controlled QUAZAR AML-001 trial. Haematologica.

